# *Helicobacter pylori* is associated with dyslipidemia but not with other risk factors of cardiovascular disease

**DOI:** 10.1038/srep38015

**Published:** 2016-11-28

**Authors:** Tae Jun Kim, Hyuk Lee, Mira Kang, Jee Eun Kim, Yoon-Ho Choi, Yang Won Min, Byung-Hoon Min, Jun Haeng Lee, Hee Jung Son, Poong-Lyul Rhee, Sun-Young Baek, Soo Hyun Ahn, Jae J. Kim

**Affiliations:** 1 Center for Health Promotion, Samsung Medical Center, Sungkyunkwan University School of Medicine, Seoul, Korea; 2 Department of Medicine, Samsung Medical Center, Sungkyunkwan University School of Medicine, Seoul, Korea; 3 Biostatistics and Clinical Epidemiology Center, Samsung Medical Center, Sungkyunkwan University School of Medicine, Seoul, Korea

## Abstract

Epidemiologic and clinical data suggest that *Helicobacter pylori* infection is a contributing factor in the progression of atherosclerosis. However, the specific cardiovascular disease risk factors associated with *H. pylori* remain unclear. We performed a cross-sectional study of 37,263 consecutive healthy subjects who underwent a routine health check-up. In multivariable log Poisson regression models adjusted for potential confounders, the associations of *H. pylori* seropositivity with higher LDL-C (relative risk [RR], 1.21; 95% confidence interval [CI], 1.12–1.30) and lower HDL-C level (RR, 1.10; 95% CI, 1.01–1.18) were significant and independent. In multiple linear regression analyses, *H. pylori* infection was significantly associated with higher total cholesterol level (coefficient = 2.114, *P* < 0.001), higher LDL-C level (coefficient = 3.339, *P* < 0.001), lower HDL-C level (coefficient = −1.237, *P* < 0.001), and higher diastolic blood pressure (coefficient = 0.539, *P* = 0.001). In contrast, *H. pylori* infection was not associated with obesity-related parameters (body mass index, waist circumference), glucose tolerance (fasting glucose, glycated hemoglobin), and systolic blood pressure. We found that *H. pylori* infection was significantly and independently associated with dyslipidemia, but not with other cardiometabolic risk factors, after adjusting for potential risk factors of atherosclerosis.

*Helicobacter pylori* colonizes the stomach of at least half the world’s population and is a key constituent of the human microbiome. Infection is usually acquired early in life and, when left untreated, persists throughout the life of the host^1^,^2^. Clinical manifestations of *H. pylori* infection include peptic ulcer disease, non-cardia gastric adenocarcinoma, and gastric mucosa-associated lymphoid tissue lymphoma. Nonetheless, most individuals with *H. pylori* infection remain asymptomatic throughout life despite chronic gastritis^1^,^3^,^4^.

Over the past few decades, a large amount of epidemiologic and clinical data regarding associations with non-gastric systemic diseases and *H. pylori* infection have been reported, including cardiovascular disease and its risk factors^5^,^6^,^7^. A number of epidemiologic studies report a significant correlation of cardiovascular disease or its risk factors with *H. pylori* infection^6^,^8^,^9^,^10^,^11^. However, the results of several other studies failed to confirm the association^12^,^13^,^14^. The inconsistent findings of these studies may be explained by varying study methodologies, such as different study population, limited sample size, or inadequate consideration of potential confounders. In particular, most previous studies did not controll for socioeconomic status, which is significantly related with prevalence of *H. pylori* infection^15^,^16^. Moreover, socioeconomic status, especially education level, is a significant predictor for cardiovascular disease and its risk factors^17^.

In addition, there are several studies regarding the role of *H. pylori* in risk factors of cardiovascular disease including type 2 diabetes, hypertension, dyslipidemia, obesity or metabolic syndrome^5^,^7^,^18^,^19^,^20^. However, only a few studies have investigated the relationships of *H. pylori* infection with each risk factor of cardiovascular disease. Therefore, we aimed to assess the association between *H. pylori* infection and each cardiometabolic risk factors in a large asymptomatic population, with control for potential confounders.

## Results

### Clinical and demographic characteristics according to *H. pylori* serostatus

Of the 37,263 subjects, 20,932 (56.2%) were men and 16,331 (43.8%) were women, with a mean age of 49.6 years. The subjects were categorized into either *H. pylori* seronegative or *H. pylori* seropositive groups; the prevalence of *H. pylori* infection was 59.0%. The overall prevalence of metabolic syndrome was 12.7% (n = 4,716). The clinical and demographic characteristics of the *H. pylori* seropositive and seronegative groups are shown in Table 1. The proportion of men was significantly higher in the *H. pylori* seropositive group. The mean age of the seropositive group was higher than the seronegative group. Heavy alcohol consumers were more likely to be seropositive. Hypertension, diabetes, and dyslipidemia were more prevalent in the seropositive group. In addition, the seropositive group was more likely to exercise regularly. The values of metabolic parameters, including body mass index (BMI), waist circumference, systolic blood pressure, diastolic blood pressure, total cholesterol, low-density lipoprotein cholesterol (LDL-C), triglycerides, fasting plasma glucose (FPG), and glycated hemoglobin (HbA1c), were significantly higher in the seropositive group; high-density lipoprotein cholesterol (HDL-C) level was significantly lower in the seropositive group. In addition, the overall prevalence of metabolic syndrome was 12.7%, and the prevalence rate of metabolic syndrome was significantly higher in the seropositive group. Clinical and demographic characteristics according to metabolic syndrome status are available in supplementary Table 1.

### Multivariable analyses of the association between *H. pylori* infection and metabolic syndrome

Possible predictors of metabolic syndrome from multivariable analysis are presented in Table 2. Factors significantly associated with the presence of metabolic syndrome included age, male sex, current smoker, BMI, body fat percentage, and alanine aminotransferase (ALT) and uric acid levels; in contrast, high education level and high income had a protective effect against metabolic syndrome. However, *H. pylori* seropositivity was not associated with the presence of metabolic syndrome.

### Multivariable analyses of the association between *H. pylori* infection and each metabolic risk factor

In examining the association of *H. pylori* infection with each metabolic risk factor, we conducted multivariable logistic regression analysis with variables selected in univariable analysis. The selected variables included age, sex, current smoker, heavy alcohol consumer, regular exercise, education level, income, BMI, body fat rate, glomerular filtration rate (GFR), ALT, uric acid, high-sensitivity C-reactive protein (HS-CRP) levels, and *H. pylori* seropositivity. As shown in Table 3, *H. pylori* seropositivity was not a risk factor for central obesity as indicated waist circumference, higher blood pressure (BP), and higher FPG. In contrast, *H. pylori* seropositivity was a significant risk factor for higher LDL-C (relative risk [RR], 1.21; 95% confidence interval [CI], 1.12–1.30; *P* < 0.001) and lower HDL-C level (RR, 1.10; 95% CI, 1.01–1.18; *P* = 0.021), but was not associated with higher triglyceride level (Table 4).

### Multivariable analyses of the relationship between *H. pylori* infection and risk factors of cardiovascular disease

**Figure 1:**
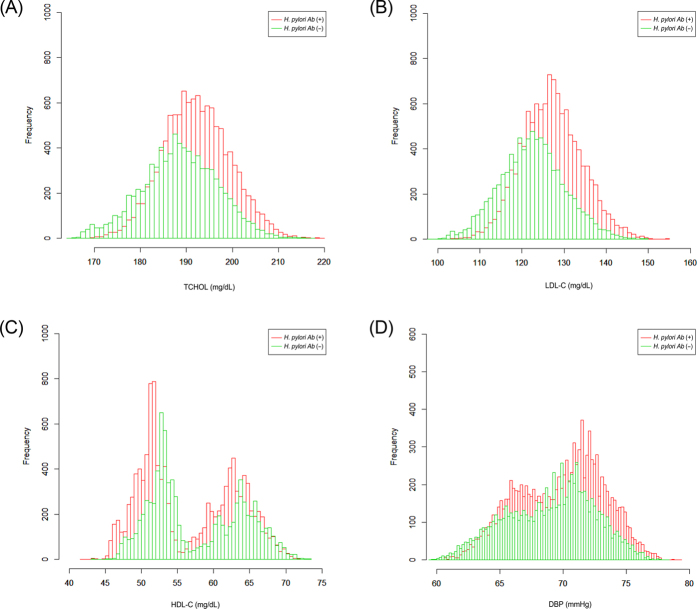
(**A**) Histogram of predicted total cholesterol by *H. pylori* status; (**B**) Histogram of predicted low-density lipoprotein cholesterol by *H. pylori* status; (**C**) Histogram of predicted high-density lipoprotein cholesterol by *H. pylori* status; (**D**) Histogram of predicted diastolic blood pressure by *H. pylori* status

The relationship of *H. pylori* seropositivity and cardiovascular risk factors were assessed by multiple linear regression analysis. In evaluating the relationship of *H. pylori* seropositivity with cardiovascular risk factors, we performed multivariable analysis after adjusting for potential confounders including age, sex, education level, income level, smoking status, alcohol consumption, and physical inactivity. *H. pylori* seropositivity showed a significant relationship with higher total cholesterol level (coefficient = 2.114, *P* < 0.001), higher LDL-C level (coefficient = 3.339, *P* < 0.001), lower HDL-C level (coefficient = −1.237, *P* < 0.001), and higher diastolic BP (coefficient = 0.539, *P* = 0.001; Fig. 1). In contrast, there was no positive relationship between *H. pylori* seropositivity and obesity-related parameters (BMI, waist circumference), glucose tolerance (FPG, HbA1c), systolic BP, and triglyceride level (Table 5).

## Discussion

In this large cross-sectional study of asymptomatic men and women undergoing routine health check-up, we investigated the association of *H. pylori* infection with risk factors for cardiovascular disease. *H. pylori* infection was a significant and independent risk factor for dyslipidemia including high LDL-C and low HDL-C levels, but not for other cardiovascular risk factors, after adjusting for potential confounders. These results support that *H. pylori* infection has a role in promoting atherosclerosis through dyslipidemia.

There is debate concerning the effect of *H. pylori* infection on obesity. A meta-analysis of 18 epidemiological studies involving a total of 10,000 subjects claimed a strong correlation between *H. pylori* infection and obesity as defined by high BMI^21^. In contrast, a recent systemic review of 49 studies with a total of 99,463 subjects demonstrated that the prevalence of *H. pylori* infection is inversely associated with the prevalence of obesity and overweight^19^. This result is consistent with recent observations from controlled trials that patients who underwent *H. pylori* eradication developed significant weight gain as compared to subjects with untreated *H. pylori* colonization^22^,^23^. One possible mechanism for significant weight gain after *H. pylori* eradication is *H. pylori*-induced increases in ghrelin, which signals hunger and appetite^24^,^25^. In the present study, *H. pylori* infection was not associated with general obesity as determined using BMI or central obesity measures such as waist circumference, after adjusting for potential confounding factors. It is interesting that central obesity, considered a core component of metabolic syndrome, was not associated with *H. pylori* infection.

The link between *H. pylori* and diabetes and glucose intolerance also remains controversial. A recent meta-analysis of 41 studies including a total of 14,080 patients revealed a higher prevalence rate of *H. pylori* in type 2 diabetes patients than in non-diabetic patients^26^. In contrast, a large well-designed study in Australia demonstrated that *H. pylori* infection was not different between diabetic and non-diabetic patients^27^,^28^. The discrepancies are likely due to adjustments for potential confounders, the method used to define diabetic status, and the limited sample sizes. In addition, the accuracy of self-reported data on diabetes depends on the subjects’ knowledge and understanding of the relevant information; thus, diabetes can be easily misclassified. In the present study, we used laboratory markers of diabetes, such as FPG and HbA1c, and conducted the analysis after controlling for all possible confounders. The present study found no association between *H. pylori* infection and glucose intolerance or diabetes mellitus.

Previous studies regarding the association of *H. pylori* infection with lipid metabolism showed relatively consistent evidence, but conflicting results also exist^5^,^7^,^18^,^29^,^30^. Especially, low socioeconomic level and crowded living conditions are important risk factors in *H. pylori* infection^15^,^16^. Also, low socioeconomic status tends to relate to an increased risk in dyslipidemia and cardiovascular disease^9^,^17^. Therefore, consideration of socioeconomic status as a potential confounding factor is important for studying the association of *H. pylori* with cardiovascular disease and its risk factors. However, most previous studies concerning the association between *H. pylori* and dyslipidemia controlled for socioeconomic status. In this study, we found that subjects with *H. pylori* infection had higher total cholesterol and LDL-C, as well as lower HDL-C, regardless of other potential confounding factors such as age, sex, socioeconomic status, BMI, smoking status, alcohol consumption, and amount of exercise. The alterations of lipid profile may be mediated by inflammatory cytokines such as interleukin-1, interleukin-6, or tumor necrosis factor-α through a chronic inflammatory condition induce by *H. pylori*^31^,^32^.

Metabolic syndrome is a multifactorial condition in which *H. pylori* infection seems to play a minor role. The prevalence of *H. pylori* infection is decreasing in developed countries; thus, it might not correspond with the recent increase in the global prevalence of metabolic syndrome or obesity-related morbidities^33^. Nevertheless, the present results elucidate the role of *H. pylori* infection in cardiovascular disease and its risk factors. Few studies have discussed the potentially different impacts that *H. pylori* infection has on each cardiovascular risk factors. Shin *et al*. compared the association between metabolic syndrome and *H. pylori* infection diagnosed by serologic and histologic status. The study found that the metabolic syndrome was more strongly associated with histologic positivity than serologic positivity. Among the cardiometabolic parameters, central obesity, low HDL-C levels, and high blood pressure were significantly associated with *H. pylori* infection after adjusting for age, sex, smoking status, alcohol consumption, and economic status^34^. In contrast, our study assessed *H. pylori* infection status solely with serologic test. However, we evaluated the association between *H. pylori* seropositivity and the cardiometabolic risk factors, paying particular attention to careful control for known risk factors and confounders including age, sex, smoking status, alcohol consumption, socioeconomic status, physical activity, and body mass index. Physical inactivity and obesity are established risk factors of metabolic syndrome and cardiovascular disease^35^,^36^,^37^. The current study provides evidence that *H. pylori* infection is associated with dyslipidemia such as higher total cholesterol and LDL-C, as well as lower HDL-C, regardless of potential confounders and putative risk factors. However, there were no evidence of the associations between *H. pylori* infection and other cardiovascular risk factors such as central obesity and glucose tolerance.

Several limitations need to be a considered in the interpretation of results. First, the evaluation of *H. pylori* infection status was done solely with serum IgG to *H. pylori* measured by enzyme-linked immunosorbent assay, without other laboratory assessments such as a rapid urease test or urease breath test. Accordingly, the possibility of false-negative or false-positive results cannot be completely excluded. However, the serum IgG antibody test to *H. pylori* is a relative highly sensitive and cheap mass screening tool that can be used easily in areas with a high prevalence of *H. pylori* infection. Second, although we measured for several important confounding factors in the multivariable analysis, we cannot exclude the possibility of residual confounders due to factors measured with error or unmeasured factors such as dietary factors.

In this large cross-sectional study, after adjusting for potential confounding factors, *H. pylori* infection was significantly and independently associated with dyslipidemia, including higher total cholesterol and LDL-C and lower HDL-C levels, but not with other cardiovascular risk factors. A larger body of evidence implies that *H. pylori* infection is a causal risk factor for cardiovascular disease; the current study provides strong evidence that *H. pylori* modifies the lipid profile that eventually promotes atherosclerosis. Eradication of *H. pylori* should be considered for patients with *H. pylori* infection and dyslipidemia, particularly in groups at high risk for cardiovascular disease.

## Methods

### Study population

We performed a cross-sectional study of healthy subjects who underwent a routine health check-up, including an *H. pylori*-specific immunoglobulin G antibody (IgG) test, at the Center for Health Promotion, Samsung Medical Center in South Korea. Regular health check-ups are very common in South Korea owing to the Industrial Safety and Health Law; the National Cancer Screening Program recommends biennial health examinations, including for several cancers^38^.

This study included 38,426 consecutive healthy subjects who underwent a health screening examination with serum IgG anti-*H. pylori* test between January 2004 and December 2007. Those who had missing data (n = 1,163; e.g., BP, FBG, LDL-C, HDL-C, triglycerides, waist circumference, and BMI) or had a history of cancer were excluded, resulting in a final sample of 37,263 subjects. This study was approved by the Institutional Review Board of the Samsung Medical Center and was conducted in accordance with the Declaration of Helsinki. The Institutional Review Board approval was obtained without specific informed consent because the study used only de-identified data that were collected for clinical purposes as part of the health screening check-up. However, informed consent was obtained from all subjects for their examinations at the health check-up.

### Data collection

The comprehensive health screening program included demographic characteristics, anthropometric data, serum biochemical measurements, and an epidemiological questionnaire assessing smoking, alcohol consumption, physical activity, education level, income, medication history, and personal medical history^39^. The personal medical histories were used to collect information regarding history of hypertension, diabetes mellitus, dyslipidemia, and cardiovascular and cerebrovascular disease. Education levels were stratified as low (elementary school or less), medium (middle or high school), or high (college or higher) and income was stratified into tertiles (lowest tertile, middle tertile, highest tertile). The medication history included current and regular use of medications for hypertension, diabetes, and dyslipidemia. Height and weight were measured in the morning to the nearest 0.1 kg and 0.1 cm, respectively; the measurements were taken with subjects wearing light clothing and barefoot. BMI was calculated as weight in kilograms divided by height in square meters (kg/m^2^), and waist circumference was measured, in a horizontal plane, half way between the lowest margin of the twelfth rib and the superior iliac crest. Body fat percentage was measured using bioelectrical impedance analysis (Inbody 720 machine, Biospace, Seoul, Korea). Systolic BP and diastolic BP were measured after the subjects rested for at least 5 minutes in a sitting position. Smoking status was assessed as never smoker, former smoker, or current smoker; alcohol consumption was assessed as never or occasionally (once or twice per month), once or twice per week, three or four times per week, or five or more times per week. Regular exercise was defined as physical activity of at least moderate intensity at least 30 minutes ≥3 days per week.

After a ≥12 hours fast, fasting blood samples were obtained from the antecubital vein, and were used to determine the serum levels of FPG, HbA1c, total cholesterol, LDL-C, and HDL-C, triglycerides, and HS-CRP. HbA1c was measured by using a high-performance liquid chromatography method with a Tosoh Glycohemoglobin Analyzer (Tosoh Bioscience Inc, Tokyo, Japan). Serum glucose was measured by using the hexokinase/glucose-6-phosphate dehydrogenase method with a Hitachi 7600 Modular Dp-110 autoanalyzer (Hitachi, Tokyo, Japan). Total cholesterol, LDL-C, HDL-C, and triglycerides were measured by using enzymatic or colorimetric methods. Serum IgG antibody to *H. pylori* was measured by an enzyme-linked immunosorbent assay, GAP test IgG kit (Bio-Rad Laboratories Inc, Hercules, Calif). *H. pylori* infection was defined as a positive enzyme-linked immunosorbent assay result.

The definition of metabolic syndrome was based on the NCEP ATP III criteria except for the cut off values of waist circumference, which were defined according to the Korean Society for the Study of Obesity^40^,^41^. Individuals with at least three of the following five traits were classified as having metabolic syndrome: (1) central obesity, defined as a waist circumference ≥90 cm for men and ≥85 cm for women; (2) high serum triglycerides, defined as ≥ mg/dL (1.7 mmol/L) or drug treatment for this lipid abnormality; (3) low HDL-C, defined as ≤40 mg/dL (1.0 mmol/L) for men and ≤50 mg/dL (1.3 mmol/L) for women or drug treatment for this lipid abnormality; (4) high BP, defined as BP ≥130/85 mmHg or drug treatment for previously diagnosed hypertension; and (5) high FPG, defined as >100 mg/dL (5.6 mmol/L) or drug treatment for previously diagnosed diabetes.

### Statistical analysis

Continuous variables are reported as means ± standard deviation, whereas categorical variables are presented as percentages. Continuous variables were compared between groups using the Wilcoxon rank sum test, whereas categorical variables were compared using the chi-squared test. The associations of *H. pylori* seropositivity with traditional risk factors of atherosclerosis and each factor of metabolic syndrome were assessed by means of relative risks (RRs) with 95% confidence intervals (CIs) using univariable and multivariable log Poisson regression analyses. Variables with *P* value <0.1 in the univariable analysis were selected for the multivariable analysis. The variables used for the multivariable analysis included age, sex, education level, income, BMI, body fat percentage, smoking status, alcohol consumption, physical activity, GFR, ALT, HS-CRP. The relationship between *H. pylori* seropositivity and each risk factor of atherosclerosis was then investigated using multiple linear regression analysis after adjusting for potential confounders including age, sex, education level, income, smoking status, alcohol consumption, and physical activity. A *P*-value <0.05 was considered statistically significant; statistical analyses were performed using SAS version 9.4 (SAS Institute, Cary, NC).

## Additional Information

**How to cite this article**: Kim, T. J. *et al. Helicobacter pylori* is associated with dyslipidemia but not with other risk factors of cardiovascular disease. *Sci. Rep.* **6**, 38015; doi: 10.1038/srep38015 (2016).

**Publisher's note:** Springer Nature remains neutral with regard to jurisdictional claims in published maps and institutional affiliations.

## Figures and Tables

**Table 1:** Baseline characteristics of individuals according to *H. pylori* status.

	*H. pylori* Ab (−)	*H. pylori* Ab (+)	*P* value
Age, years	48.4 ± 10.8	50.4 ± 9.5	<0.001
Sex			<0.001
Male	8,292 (54.3)	12,640 (57.5)	
Female	6,986 (45.7)	9,346 (42.5)	
Smoking status			<0.001
Never	7,800 (51.1)	10,951 (49.8)	
Former	937 (6.1)	1,571 (7.2)	
Current	3,196 (20.9)	4,154 (18.9)	
Alcohol consumption			0.012
Never or occasional	8,888 (58.2)	12,359 (56.2)	
Once or twice a week	2,945 (19.3)	4,341 (19.7)	
Three or four times a week	1,510 (9.9)	2,320 (10.6)	
Five or more times a week	573 (3.8)	854 (3.9)	
Physical activity			0.026
Two or less times a week	8,245 (54.0)	11,623 (52.9)	
Three or four times a week	6,281 (41.1)	9,296 (42.3)	
History of hypertension	2,397 (15.7)	3,705 (16.9)	0.003
History of diabetes	832 (5.5)	1,355 (6.2)	0.004
History of dyslipidemia	1,760 (11.5)	2,790 (12.7)	0.001
History of angina or myocardial infarction	339 (2.2)	514 (2.3)	0.451
History of stroke	154 (1.0)	235 (1.1)	0.57
Body fat percentage (%)	23.9 ± 6.2	23.9 ± 6.3	0.427
BMI (kg/m^2^)	23.6 ± 3.0	23.8 ± 2.9	<0.001
Waist circumference (cm)	81.9 ± 9.7	82.8 ± 9.3	<0.001
SBP (mmHg)	113.9 ± 15.6	114.9 ± 15.8	<0.001
DBP (mmHg)	69.3 ± 10.5	70.2 ± 10.6	<0.001
TG (mg/dL)	126.5 ± 79.6	128.4 ± 77.6	<0.001
HDL-C (mg/dL)	57.9 ± 14.4	56.5 ± 13.8	<0.001
LDL-C (mg/dL)	123.6 ± 31.2	127.6 ± 30.7	<0.001
TCHOL (mg/dL)	190.1 ± 34.0	192.7 ± 33.4	<0.001
FPG (mg/dL)	91.8 ± 17.4	92.3 ± 17.4	<0.001
HbA1c (%)	5.4 ± 0.7	5.5 ± 0.7	<0.001
GFR (mL/min)	90.5 ± 13.8	88.6 ± 13.1	<0.001
AST (U/L)	23.9 ± 13.1	24.1 ± 12.1	<0.001
ALT (U/L)	24.2 ± 19.6	24.5 ± 20.0	<0.001
Uric acid (mg/dL)	5.2 ± 1.4	5.3 ± 1.4	<0.001
HS-CRP (mg/dL)	0.16 ± 0.43	0.16 ± 0.47	0.1
Metabolic syndrome, n (%)	1,837 (12.0)	2,880 (13.1)	0.002

Variables are expressed as n (%) or mean ± SD.

*H. pylori, Helicobacter pylori*; Ab, antibody; BMI, body mass index; SBP, systolic blood pressure; DBP, diastolic blood pressure; TG, triglycerides; FPG, fasting plasma glucose; HDL-C, high-density lipoprotein cholesterol; LDL-C, low-density lipoprotein cholesterol; TCHOL, total cholesterol; HbA1c, glycated hemoglobin; GFR, glomerular filtration rate; AST, aspartate aminotransferase; ALT, alanine aminotransferase; HS-CRP, high sensitivity C-reactive protein, SD, standard deviation.

**Table 2:** Association between *Helicobacter pylori* infection and metabolic syndrome.

	Multivariable analysis		
	RR	95% CI	*P* value
Age	1.04	1.03–1.08	<0.001
Male sex	1.55	1.22–1.96	<0.001
Current smoker	1.59	1.41–1.79	<0.001
Alcohol consumption	1.10	0.90–1.32	0.372
Regular exercise	0.96	0.87–1.05	0.389
High education level	0.84	0.73–0.96	0.014
High income	0.86	0.74–0.99	0.046
BMI	1.23	1.20–1.25	<0.001
Body fat percentage	1.02	1.01–1.04	<0.001
GFR	1.00	0.99–1.01	0.954
ALT	1.01	1.01–1.02	<0.001
Uric acid	1.08	1.04–1.12	<0.001
HS-CRP	1.02	0.94–1.11	0.591
*H. pylori*	1.02	0.93–1.11	0.707

Relative risks ± 95% CI were assessed by multivariate log Poisson analysis.

RR, relative risk; CI, confidence interval; BMI, body mass index; GFR, glomerular filtration rate; ALT, alanine aminotransferase; HS-CRP, high sensitivity C-reactive protein; *H. pylori, Helicobacter pylori*.

**Table 3:** Association of *Helicobacter pylori* infection with metabolic risk factors except for the lipid profile.

	Elevated WC		Elevated BP		Elevated FPG	
	RR (95% CI)	*P* value	RR (95% CI)	*P* value	RR (95% CI)	*P* value
Age	1.02 (1.01–1.02)	<0.001	1.04 (1.04–1.05)	<0.001	1.05 (1.04–1.06)	<0.001
Male sex	2.25 (1.86–2.72)	<0.001	1.27 (1.05–1.53)	0.014	1.61 (1.29–1.99)	<0.001
Current smoker	1.23 (1.13–1.34)	<0.001	1.01 (0.88–1.17)	0.859	1.13 (1.02–1.25)	0.025
Alcohol consumption	1.16 (1.01–1.35)	0.046	1.26 (1.06–1.49)	0.009	1.52 (1.30–1.79)	<0.001
Regular exercise	0.98 (0.92–1.05)	0.684	1.08 (0.95–1.20)	0.454	1.02 (0.93–1.12)	0.406
High education level	0.89 (0.79–0.98)	0.023	0.89 (0.79–1.00)	0.05	0.99 (0.87–1.12)	0.839
High income	0.84 (0.74–0.94)	0.003	0.91 (0.79–1.02)	0.127	1.01 (0.88–1.17)	0.852
BMI	1.24 (1.22–1.26)	<0.001	1.09 (1.07–1.11)	<0.001	1.11 (1.08–1.13)	<0.001
Body fat percentage	1.04 (1.03–1.05)	<0.001	1.02 (1.01–1.03)	<0.001	1.01 (0.99–1.02)	0.178
GFR	0.99 (0.99–1.01)	0.552	0.99 (0.99–1.01)	0.10	1.00 (0.99–1.01)	0.362
ALT	1.00 (1.00–1.01)	0.061	1.01 (1.01–1.02)	0.004	1.01 (1.01–1.02)	<0.001
Uric acid	1.04 (1.01–1.07)	0.003	1.09 (1.06–1.13)	<0.001	1.02 (1.01–1.02)	<0.001
HS-CRP	1.01 (0.95–1.07)	0.823	1.03 (0.96–1.09)	0.428	1.06 (1.01–1.12)	0.036
*H. pylori*	1.06 (0.99–1.13)	0.094	1.06 (0.98–1.14)	0.125	0.98 (0.91–1.06)	0.653

Relative risks ± 95% CI were assessed by multivariate log Poisson analysis.

WC, waist circumference; BP, blood pressure; FPG, fasting plasma glucose; RR, relative risk; CI, confidence interval; BMI, body mass index; GFR, glomerular filtration rate; ALT, alanine aminotransferase; HS-CRP, high sensitivity C-reactive protein; *H. pylori, helicobacter pylori*.

**Table 4:** Association of *Helicobacter pylori* infection with risk of dyslipidemia-related variables.

	High LDL-C		Low HDL-C		High TG	
	RR (95% CI)	*P* value	RR (95% CI)	*P* value	RR (95% CI)	*P* value
Age	1.01 (1.00–1.02)	0.012	1.01 (1.01–1.02)	<0.001	1.02 (1.02–1.03)	<0.001
Male sex	1.07 (0.85–1.34)	0.592	0.57 (0.47–0.70)	<0.001	1.50 (1.27–1.77)	<0.001
Current smoker	0.90 (0.75–1.09)	0.269	1.64 (1.45–1.84)	<0.001	1.55 (1.43–1.67)	<0.001
Alcohol consumption	1.01 (0.92–1.12)	0.799	0.56 (0.42–0.74)	<0.001	1.07 (0.98–1.17)	0.159
Regular exercise	0.99 (0.94–1.06)	0.886	0.85 (0.79–0.92)	<0.001	0.85 (0.80–0.90)	<0.001
High education level	0.86 (0.75–0.98)	0.002	0.81 (0.72–0.92)	0.001	0.79 (0.72–0.86)	<0.001
High income	0.92 (0.83–1.02)	0.146	1.04 (0.91–1.20)	0.528	1.01 (0.92–1.09)	0.929
BMI	1.06 (1.04–1.08)	<0.001	1.06 (1.04–1.08)	<0.001	1.10 (1.08–1.12)	<0.001
Body fat percentage	1.02 (1.01–1.03)	0.008	1.03 (1.02–1.04)	<0.001	1.01 (1.00–1.02)	0.016
GFR	1.01 (0.97–1.04)	0.568	1.00 (0.99–1.01)	0.877	1.01 (0.99–1.01)	0.113
ALT	1.02 (1.01–1.02)	<0.001	1.01 (1.01–1.02)	<0.001	1.01 (1.00–1.02)	<0.001
Uric acid	1.09 (1.05–1.13)	<0.001	1.01 (0.99–1.03)	0.308	1.11 (1.09–1.14)	<0.001
HS-CRP	0.98 (0.92–1.06)	0.715	1.09 (1.04–1.14)	<0.001	1.00 (0.96–1.05)	0.925
*H. pylori*	1.21 (1.12–1.30)	<0.001	1.10 (1.01–1.18)	0.021	1.03 (0.99–1.07)	0.22

Relative risks ± 95% CI were assessed by multivariate log Poisson analysis.

LDL-C, low-density lipoprotein cholesterol; HDL-C, high-density lipoprotein cholesterol; TG, triglycerides; RR, relative risk; CI, confidence interval; BMI, body mass index; GFR, glomerular filtration rate; ALT, alanine aminotransferase; HS-CRP, high sensitivity C-reactive protein; *H. pylori, Helicobacter pylori*.

**Table 5:** The relationship between *Helicobacter pylori* infection and risk factors of cardiovascular disease.

	Coefficient	Standard error	*P* value
BMI	0.074	0.04	0.064
Waist circumference	0.195	0.114	0.058
SBP	0.293	0.218	0.179
DBP	0.539	0.154	0.001
FPG	−0.640	0.436	0.142
HbA1c	−0.013	0.009	0.159
TCHOL	2.114	0.484	<0.001
LDL-C	3.339	0.439	<0.001
HDL-C	−1.237	0.189	<0.001
TG	0.65	1.068	0.543

Coefficient was assessed by multivariable linear regression analysis after adjusting for potential confounders including age, sex, smoking status, alcohol consumption, physical activity, education level and income level.

BMI, body mass index; SBP, systolic blood pressure; DBP, diastolic blood pressure; FPG, fasting plasma glucose; HbA1c, glycated hemoglobin; TCHOL, total cholesterol; LDL-C, low-density lipoprotein cholesterol; HDL-C, high-density lipoprotein cholesterol; TG, triglycerides.
